# Association between serum cystatin C level and cognition in older adults: a cross-sectional analysis

**DOI:** 10.3389/fnins.2023.1200763

**Published:** 2023-05-03

**Authors:** Shuli Wang, Xuechun Lin, Jie Zhou, Meng Li, Dan Song

**Affiliations:** ^1^Department of Radiology, Shandong Provincial Hospital Affiliated to Shandong First Medical University, Jinan, Shandong, China; ^2^Department of Nutrition and Food Hygiene, Hubei Key Laboratory of Food Nutrition and Safety, Ministry of Education Key Laboratory of Environment and Health, School of Public Health, Tongji Medical College, Huazhong University of Science and Technology, Wuhan, China; ^3^Department of Nursing, Shenzhen Qianhai Shekou Free Trade Zone Hospital, Shenzhen, Guangdong, China; ^4^Kentucky STD Prevention and Control, Frankfort, KY, United States

**Keywords:** biomarker, cystatin C, psychomotor test, gerontology, national survey, kidney function

## Abstract

**Introduction:**

Serum Cystatin C level, an indication of kidney function, has been implicated in the pathogenesis of Alzheimer’s disease and cognitive impairment. In this cross-sectional study, we looked into the relation between serum Cystatin C levels and cognition in a group of U.S. older adults.

**Method:**

The data of this study were from the National Health and Nutrition Examination Survey (NHANES) 1999–2002. A total of 4,832 older adults aged ≥60 who met the inclusion criteria were included. The Dade Behring N Latex Cystatin C assay, which is a particle-enhanced nephelometric assay (PENIA), was utilized to assess Cystatin C levels in participants’ blood samples. Participants’ cognition was examined using the digit symbol substitution test (DSST). *Z*-scores of the DSST were calculated based on sample means and standard deviations (SD). To investigate the relationships between the quartiles of serum Cystatin C level and DSST *z* scores, multiple linear regression models were developed while controlling for age, sex, race/ethnicity, and education.

**Results:**

The average age of the participants was 71.1 (SD 7.8). The participants were about half female (50.5%), non-Hispanic White (61.2%), and (36.1%) who had completed at least some college. They had an average serum Cystatin C level of 1.0 mg/dl (SD 0.44). After performing multiple linear regression with a reference group consisting of participants in quartile one of plasma Cystatin C levels, we found that serum Cystatin C levels in quartiles three and four were independently associated with lower DSST *z* scores (β = −0.059, 95% CI −0.200 to −0.074 and β = −0.108, 95% CI −0.319 to −0.184, respectively).

**Conclusion:**

Higher serum Cystatin C level is associated with worse processing speed, sustained attention, and working memory in older adults. Cystatin C level may be a biomarker for cognitive decline in older adults.

## Introduction

The global population is aging dramatically. With the proportion of the older population expanding, promoting the health and independence of older adults is an ongoing challenge to families and society. Being old is associated with a decrease in cognitive health that ultimately impairs living independence and quality of life (Song et al., 2019). A comprehensive understanding of the potential risk factors and biomarkers of cognitive deterioration among older adults is of utmost importance to help to screen high-risk individuals and determine strategies to alleviate these effects on the population and reduce the burden of cognitive impairment on individuals and society.

A group of socio-demographic factors such as older age and lower education and psychosocial factors (such as depressive symptoms and social isolation) have been established as risk factors for cognitive decline in older adults (Song et al., 2018; Warren and Bamiou, 2018; Livingston et al., 2020). Biomarkers of cognitive decline in the older population are not fully studied. Cystatin C has received increasing attention as a potential biomarker for cognitive impairment in the older population because studies found that cystatin C colocalizes with β-amyloid in the brains of individuals with Alzheimer’s disease (AD; Levy et al., 2001; Sastre et al., 2004). Cystatin C is a protein that belongs to a group of cysteine proteases inhibitors generated mainly by nucleated cells (Mussap and Plebani, 2004). The serum concentration of cystatin C is independent of gender and age; therefore, it has been widely studied as an endogenous marker of glomerular filtration rate (Filler et al., 2005). In addition to its role as a marker for renal function, cystatin C plays various biological roles, ranging from cell proliferation and growth to modulating potentially pathogenic events, including neurodegenerative disorders (Mathews and Levy, 2016). Cystatin C is linked to the risk of cognitive impairment through genetic and neuropathological pathways. Genetically, the CST3 B haplotype of cystatin C was considered to be a risk factor for AD, frontotemporal dementia, and Lewy body dementia (Finckh et al., 2000; Benussi et al., 2010; Maetzler et al., 2010). In addition, cystatin C colocalizes with β-amyloid in the brain, especially in areas involved in AD pathology (Sastre et al., 2004).

The association between Cystatin C levels and cognitive impairment among general older adults has revealed different results in previous studies (Sundelöf et al., 2008; Wang et al., 2017; Zhang et al., 2019; Chen et al., 2021). A recent review conducted a subgroup analysis based on ethnicity and revealed that the increased level of Cystatin C was associated with the risk of cognitive impairment in the Asian population but not in the Caucasian population (Nair et al., 2020). Furthermore, the association between cystatin C and cognitive decline has not been fully investigated in community-based older adults.

Therefore, the aim of the present study is to investigate the relationship between cystatin C level and cognitive function in a nationally representative group of older participants in the National Health and Nutrition Examination Survey (NHANES) 2000–2002. The findings of this study will help elucidate the effect of cystatin C on cognitive function among older adults, which has significant benefits in diagnostic as well as therapeutic implications for cognitive impairment among older adults.

## Methods

### The parent study design

The NHANES program is a biannual series of continuous cross-sectional surveys designed to assess the health and nutrition of people in the USA. It is administered by the National Center for Health Statistics, which is part of the Centers for Disease Control and Prevention. The program has evolved to encompass a wide range of health and nutrition measurements. To ensure that the data collected is representative of the civilian, noninstitutionalized population, the NHANES employs a complex, multistage probability design instead of a simple random sampling method (Johnson et al., 2013). A team of qualified doctors, technicians, and interviewers performed a range of measurements to evaluate the demographic, socioeconomic, nutrition, and health-related status of the participants. In-person interviews and physical examinations were conducted at participants’ residences and mobile exam centers, respectively, with specialized settings. The inclusion criteria for this study were being at least 60 years old and having information available on both serum Cystatin C level and cognition. Data from the NHANES 1999–2000 (*n* = 25,232) and 2001–2002 cycles (*n* = 24,896) were combined. We excluded those under 60 years old (*n* = 44,737), those without available information on serum Cystatin C level (*n* = 559), and those without cognitive performance data (*n* = 0). The final sample for this study consisted of 4,832 older adults aged 60 years and above.

### Ethical considerations

The NHANES study was granted approval by the Research Ethics Review Board of the National Center for Health Statistics. Informed written consent was obtained from all participants prior to their enrollment in the study.

### Measures

#### Independent variable: quartile of serum cystatin C level [quartile one (reference group), quartile two, quartile three, and quartile four]

The Dade Behring N Latex Cystatin C assay is a particle-enhanced nephelometric assay (PENIA). It was utilized to assess Cystatin C levels in blood samples. This assay was performed on the Dade Behring Nephelometer II (BNII; Finney et al., 1997). According to Newman’s (2002) assessment of various assay methodologies, the current assay is the most accurate and precise among automated assays across the clinical concentration range, with an intra-assay imprecision range between 2.0 and 3.0% coefficient of variation. The inter-assay imprecision range is between 3.2 and 4.4% coefficient of variation. Additionally, the assay range is between 0.23 and 7.25 mg/dl. Newman also found that this assay had superior sensitivity and lacked analytical interference compared to other automated assays. The participants’ serum Cystatin C level was categorized into four groups (quartile one, quartile two, quartile three, and quartile four).

#### Dependent variable: cognitive function

In NHANES 1999–2002, with the Digit Symbol Substitution Test (DDST), participants’ processing speed, sustained attention, and working memory were examined (Ryan and Schnakenberg-Ott, 2003). Two experienced interviewers in a mobile physical examination center conducted all of the questionnaires on the same day. The surveys gave participants the option of taking them in their favorite language. The DSST is a cognitive test that uses paper and pencil and is given on a single sheet of paper. Participants must match symbols with numbers using a key at the top of the page (Jaeger, 2018). The DSST required participants to draw symbols in one hundred thirty-three numbered boxes based on a provided key. The score for the test was calculated based on the number of correct matches, which could range from 0 to 133. This test has been utilized widely in public health research studies (Plassman et al., 2007; Proust-Lima et al., 2007; Rosano et al., 2016).

#### Covariates

In order to reduce the possible confounding impact of serum Cystatin C levels on cognition, we accounted for several variables, including age (in years), gender (male or female), race/ethnicity (Mexican Americans, other Hispanics, non-Hispanic White, or non-Hispanic Black), and education (below high school, high school graduate, or some college or above).

### Statistical analysis

Descriptive statistics were used to summarize the data. Means and standard deviations (SD) were used for continuous data, while frequency and percentages were used for categorical data. To investigate the independent association between serum Cystatin C level quartile and cognition, we developed a multivariate linear regression model while controlling for the previously mentioned confounding variables. The independent variable was Cystatin C quartile (with quartile one as the reference) and the dependent variable was the DSST *z* scores. Before constructing the regression model, we evaluated multicollinearity among the covariates, with no multicollinearity being observed (variance inflation factor < 10). We set statistical significance at a 95% confidence interval (CI) that excluded zero. All analyses were performed using SPSS 25.0.

## Results

Table 1 showed the sociodemographic and health characteristics of the participants. Of the 4,832 participants (average age 71.1, standard deviation [SD] 7.8). Roughly half were female (50.5%), non-Hispanic White (61.2%), and completed some college or above (36.1%). Their mean plasma Cystatin C level was 1.0 μg/dl (SD 0.44). Their mean DSST score was 41.9 (SD 18.6).

**Table 1:** The participants’ characteristics, total and by serum Cystatin C level quartile.^a^

Variables	Quartile 1 ≤ 0.786 mg/dl (*n* = 1,214)	Quartile 2 0.786–0.903 mg/dl (*n* = 1,204)	Quartile 3 0.903–1.07 mg/dl (*n* = 1,220)	Quartile 4 > 1.07 mg/dl (*n* = 1,194)	Total (*n* = 4,832)
Age, years	67.1 (6.1)	69.2 (6.8)	72.1 (7.5)	76.3 (7.4)	71.1 (7.8)
Sex, *n* (%)					
Male	534 (44.0%)	574 (47.7%)	650 (53.3%)	634 (53.1%)	2,392 (49.5%)
Female	680 (56.0%)	630 (52.3%)	570 (46.7%)	560 (46.9%)	2,440 (50.5%)
Race/ethnicity, *n* (%)					
Hispanics and others	446 (36.7%)	346 (28.7%)	248 (20.3%)	182 (15.2%)	1,222 (25.3%)
Non-Hispanic Whites	544 (44.8%)	714 (59.3%)	800 (65.6%)	900 (75.4%)	2,958 (61.2%)
Non-Hispanic Blacks	224 (18.5%)	144 (12.0%)	172 (14.1%)	112 (9.4%)	652 (13.5%)
Education, *n* (%)					
Below high school	490 (40.4%)	456 (38.0%)	470 (38.6%)	494 (41.4%)	1,910 (39.6%)
High school graduate	270 (22.3%)	300 (25.0%)	296 (24.3%)	308 (25.8%)	1,174 (24.3%)
College graduate or above	452 (37.3%)	444 (37.0%)	452 (37.1%)	392 (32.8%)	1,740 (36.1%)
Serum Cystatin C level, mg/dl	0.7 (0.07)	0.8 (0.03)	1.0 (0.05)	1.4 (0.70)	1.0 (0.44)
Digit symbol substitution test	44.9 (19.4)	45.3 (18.1)	40.9 (18.6)	36.3 (16.7)	41.9 (18.6)

a The data for continuous variables were presented as mean (standard deviation), while categorical variables were presented as *n* (%).

Table 2 showed the averages and 95% CIs of the cognitive test-specific *z* scores stratified by plasma Cystatin C level quartiles. For participants in quartile one of plasma Cystatin C level, their average DSST *z* score was 0.16 (95% CI 0.10, 0.22). For participants in quartile two, their average DSST *z* score was 0.18 (95% CI −0.12, 0.23). For participants in quartile three, their average DSST *z* score was −0.05 (95% CI −0.11, 0.004). The average DSST *z* score was −0.30 (95% CI −0.30, −0.25) among participants in quartile four.

**Table 2:** The DSST *z*-scores and their corresponding 95% confidence intervals were stratified by quartiles of serum Cystatin C levels.

	Quartile 1 ≤ 0.786 mg/dl (*n* = 1,214)	Quartile 2 0.786–0.903 mg/dl (*n* = 1,204)	Quartile 3 0.903–1.07 mg/dl (*n* = 1,220)	Quartile 4 > 1.07 mg/dl (*n* = 1,194)
Digit symbol substitution test	0.16 (0.10,0.22)	0.18 (0.12,0.23)	−0.05 (−0.11,0.004)	−0.30 (−0.35,-0.25)

After performing multiple linear regression with a reference group consisting of participants in quartile one of plasma Cystatin C levels, we found that serum Cystatin C levels in quartiles three and quartile four were independently associated with lower DSST *z* scores (β = −0.059, 95% CI −0.200 to −0.074 and β = −0.108, 95% CI −0.319 to −0.184, respectively), as demonstrated in Table 3.

**Table 3:** Independent associations between quartiles of serum Cystatin C levels (reference: ≤0.786 mg/dl) and DSST *z*-scores.^a^

	Quartile 1 ≤ 0.786 mg/dl (*n* = 1,214)	Quartile 2 0.786–0.903 mg/dl (*n* = 1,204)	Quartile 3 0.903–1.07 mg/dl (*n* = 1,220)	Quartile 4 > 1.07 mg/dl (*n* = 1,194)
Digit symbol substitution test	Reference	0.005(−0.056, 0.066)	**−0.059 (−0.200, −0.074)**	**−0.108 (−0.319, −0.184)**

Bolded values mean statistical significance (95% confidence interval excluding zero).

a Age, sex, race/ethnicity, and education were adjusted in the model.

## Discussions

Our finding is that higher serum Cystatin C level is associated with worse processing speed, sustained attention, and working memory in older adults as measured by DSST *z* scores. The findings of this study indicate that Cystatin C level may be a biomarker for cognitive decline in older adults.

Cystatin C is a protein commonly utilized as a biomarker for assessing kidney function and Alzheimer’s disease (Shlipak et al., 2013). Upon its synthesis by nucleated cells, Cystatin C is secreted into the bloodstream and participates in various neurological pathways, such as suppressing the production of cysteine protease, reducing Amyloid-beta accumulation, and regulating the generation of neural cells (Mathews and Levy, 2016). Recent research has explored the relationship between Cystatin C levels and cognitive health, with some studies indicating that elevated levels of Cystatin C in the cerebrospinal fluid (CSF) may be linked to a higher likelihood of cognitive decline and dementia in older adults (Yaffe et al., 2014). Additionally, there have been reports suggesting an association between the levels of Cystatin C in the blood and the risk of developing dementia, as well as cognitive impairment (Sarnak et al., 2008; Maetzler et al., 2010).

The precise mechanism underlying the association between Cystatin C levels and cognitive function remains under investigation. Several hypotheses have been proposed in studies exploring the potential pathogenic role of Cystatin C levels in cognitive diseases. One proposed hypothesis posits that amyloid plaques, which are a hallmark of Alzheimer’s disease, are primarily composed of the protein amyloid beta. In this context, Cystatin C belongs to a type of cysteine proteinase inhibitor, which functions by binding to enzymes and reducing their ability to degrade amyloid beta protein (Murphy and LeVine, 2010; Mathews and Levy, 2016). By inhibiting the degradation of Amyloid-beta, Cystatin C can potentially prevent the accumulation of toxic Amyloid-beta aggregates in the brain, which may contribute to the preservation of cognitive function (Mi et al., 2007). Another hypothesis is the anti-inflammatory properties of Cystatin C, which have demonstrated the ability to regulate immune response and mitigate oxidative stress, thus aiding in the prevention of cognitive decline (Xu et al., 2015). Cystatin C also has the potential to affect cognitive function by modulating synaptic plasticity involved in the formation of memory (Sun et al., 2008).

This study has several strengths. Firstly, the study population is a nationally representative sample of older adults, enhancing the generalizability of the findings. Secondly, this study focuses on a vulnerable group of people, older adults who are at high risk of cognitive impairment. However, the major weakness of this study is its cross-sectional design. Therefore, we cannot establish causality or determine changes in plasma Cystatin C level and cognition over time. Third, certain confounders, such as depressive symptoms, may exist in this study. However, depressive symptoms were not measured in NHANES 1999–2002. Thus, we could not adjust depressive symptoms in the analysis. Moreover, plasma Cystatin C level with a half-life of about 1.5 h (Sjöström et al., 2004) only reflects recent levels and may not accurately reflect long-term kidney function. Furthermore, we only assessed one cognitive domain, which may limit the scope of the findings. Since the data were collected between 1999 and 2002, they might be outdated and did not reflect older adults’ current Cystatin C level and cognition. Finally, the NHANES survey may not be fully representative of certain sub-populations, such as rural populations, homeless individuals, and non-English speakers. Future studies should consider using longitudinal designs to investigate the relationship between plasma cystatin level and cognitive function across all domains in older adults, particularly those in non-western countries.

To conclude, in this study, we found a negative association between serum Cystatin C level and cognitive function in a large sample of older adults. Our finding indicates that Cystatin C level may serve as a biomarker for cognitive decline in older adults.

## Data availability statement

The original contributions presented in the study are included in the article/supplementary material, further inquiries can be directed to the corresponding author.

## Ethics statement

The studies involving human participants were reviewed and approved by the NHANES has been approved by the National Center for Health Statistics Research Ethics Review Board. The patients/participants provided their written informed consent to participate in this study. Written informed consent was obtained from the individual(s) for the publication of any potentially identifiable images or data included in this article.

## Author contributions

SW, ML, and DS designed this study, drafted the initial manuscript, and searched for literature. XL and JZ conducted the statistical analysis. All authors contributed to the article and approved the submitted version.

## Conflict of interest

The authors declare that the research was conducted in the absence of any commercial or financial relationships that could be construed as a potential conflict of interest.

## Publisher’s note

All claims expressed in this article are solely those of the authors and do not necessarily represent those of their affiliated organizations, or those of the publisher, the editors and the reviewers. Any product that may be evaluated in this article, or claim that may be made by its manufacturer, is not guaranteed or endorsed by the publisher.
